# A pharmacokinetic study on red ginseng with furosemide in equine

**DOI:** 10.3389/fvets.2023.1319998

**Published:** 2023-11-24

**Authors:** Young Beom Kwak, Eunkyu Lee, Hyunjoo Choi, Taemook Park, Ahram Kim, Jungon Kim, Jungho Yoon, Hye Hyun Yoo

**Affiliations:** ^1^Racing Laboratory, Korea Racing Authority, Jeju-si, Jeju, Republic of Korea; ^2^Institute of Pharmaceutical Science and Technology and College of Pharmacy, Hanyang University, Ansan, Republic of Korea; ^3^Training Support Team, Jeju Stud Farm, Korea Racing Authority, Jeju-si, Jeju, Republic of Korea; ^4^Equine Clinic, Korea Racing Authority, Jeju-si, Jeju, Republic of Korea; ^5^Equine Clinic, Jeju Stud Farm, Korea Racing Authority, Jeju-si, Jeju, Republic of Korea; ^6^Management Team, Jeju Stud Farm, Korea Racing Authority, Jeju-si, Jeju, Republic of Korea

**Keywords:** red ginseng, furosemide, herb-drug interaction, equine, pharmacokinetics

## Abstract

Red ginseng (RG) is a popular ingredient in traditional Korean medicine that has various health benefits. It is commonly taken orally as a dietary supplement; however, its potential interactions with concomitantly administered drugs are unclear. In this study, we examined the pharmacokinetic interaction between furosemide and RG in equine plasma. Liquid chromatography with tandem mass spectrometry analysis was performed to evaluate ginsenosides in the plasma of horses after feeding them RG and furosemide and validate the results. A single bolus of furosemide (0.5 mg/kg) was administered intravenously to female horses that had consumed RG (600 mg/kg/day) every morning for 3 weeks (experimental group), and blood samples were collected from 0 to 24 h, analyzed, and compared with those from female horses that did not consume RG (control group). Four (20s)-protopanaxadiol ginsenosides (Rb1, Rb2, Rc, and Rd) were detected in the plasma. Rb1 and Rc individually showed a high concentration distribution in the plasma. The C_max_, AUC_0−t_, and AUC_0−∞_ of furosemide was significantly increased in the experimental group (*p* < 0.05), while the CL, V_z_, and V_ss_ was decreased (*p* < 0.05, *p* < 0.01). These changes indicate the potential for pharmacokinetic interactions between furosemide and RG.

## Introduction

Red ginseng (RG), also known as Asian ginseng, is a type of ginseng (*Panax ginseng* Meyer) that has been processed through steaming and drying (1–3). This processing method is believed to enhance the medicinal properties of ginseng, making it a popular ingredient in traditional Korean medicine (4). RG has several health benefits, including enhanced energy and immunity, reduced stress and fatigue, and improved sexual function (5–7). Some studies have suggested that RG may have anti-inflammatory and antioxidant effects (8, 9). The main bioactive components of RG include ginsenosides which consist of steroidal saponins, polysaccharides with anti-inflammatory and immune-boosting properties, flavonoids, and volatile oils (3, 10–13). Based on their aglycone moieties, ginsenosides are classified into (20s)-protopanaxadiol (PPD) and (20s)-protopanaxatriol (PPT). The representative PPD ginsenosides include Rb1, Rb2, Rc, Rd., compound K, Rg3, F2, and Rh2, while the PPT ginsenosides include Rg1, Rh1, and Re (Supplementary Figure S1) (2, 14).

Furosemide (Supplementary Figure S1) is a loop diuretic, which is a class of medications that increase the amount of salt and water excreted from the body as urine (15). Loop diuretics inhibit the reabsorption of sodium and chloride ions in the loop of Henle in the kidney, leading to increased excretion of water, sodium, potassium, and other ions (16). This increased excretion results in increased urine output and decreased blood volume, leading to reduced blood pressure and improved cardiac function under certain conditions (17). Furosemide has been experimentally used in the racing industry. For years, it has been legally approved for use in the US racing industry to control exercise-induced pulmonary hemorrhage (EIPH) or bleeding. Nevertheless, its use has been criticized by organizations both inside and outside the racing industry (18). Because various drug–drug interactions with furosemide have been reported to date. Furosemide–digoxin interaction increases the risk of digitalis toxicity, and co-administration of furosemide with non-steroidal anti-inflammatory drugs increases the risk of kidney problems (19–21).

Many herbs are commonly taken orally in the form of capsules, tablets, extracts, or teas in daily life (22). Among them, RG is commonly consumed as a dietary supplement and herbal remedy (23). Interactions between ginseng and loop diuretics, particularly furosemide, have been reported in case studies. Becker et al. reported diuretic resistance to furosemide in patients who regularly consumed ginseng and cautioned against the use of furosemide in such patients (8). However, related pharmacokinetic interaction studies have not yet been reported. Therefore, it is necessary to evaluate potential RG–drug interactions in disease treatment, especially through pharmacokinetic study (24).

The purpose of this study was to evaluate the pharmacokinetics of furosemide following RG intake. The distribution of ginsenosides in equine plasma from the RG intake group was measured to confirm the presence of bioactive compounds therein after RG administration. LC–MS/MS was used to quantify ginsenosides and furosemide. The results of this analytical method were then validated. Furthermore, the pharmacokinetic parameters of furosemide in plasma were calculated and compared.

## Materials and methods

Materials, sample preparation, and validation method are described in the Supplementary data. The method was evaluated according to the guidelines of the United States Food and Drug Administration for Industry Bioanalytical Method Validation (25).

### LC–MS/MS instrumentation and conditions

Analytes were quantified using a 1,290 infinity UHPLC system and 6,470 Triple Quadrupole LC–MS/MS system (Agilent Technologies, Santa Clara, CA, United States) with electrospray ionization. The analytical column used was an Acquity UPLC high-strength-silica (HSS) C18 column (2.1 × 50 mm, 1.7 μm, Milford, MA, United States). The column temperature was maintained at 40°C, and the mobile phase consisted of 0.1% formic acid (FA) in distilled water (DW) and 0.1% FA in acetonitrile (ACN). The following gradient program was implemented: 10% B for 0.5 min, followed by a change to 60% B over 0.1 min and then to 90% B over 1 min, maintenance at 90% B for 0.5 min, returning to 10% B over 0.1 min, and finally maintenance at 10% B for 3.5 min. The flow rate was 0.2 mL/min, and the injection volume was 10 μL. Electrospray ionization was performed using nitrogen as the positive and negative capillary, with optimal settings of 3,000 V for the positive capillary, 2,500 V for the negative capillary, gas temperature of 350°C, gas flow of 10 L/min, nebulizer pressure of 12 psi, sheath gas temperature of 320°C, and sheath gas flow of 8 L/min. Multiple reaction monitoring (MRM) was performed using specified precursor–product ion pairs. Data acquisition and processing were controlled by Agilent MassHunter Data Acquisition and Qualitative Analysis software (version 10.0).

### Pharmacokinetic study in horses

All animal procedures were approved by the Korea Racing Authority (AEC-2307). Eight thoroughbred horses (female, 3–20 years, 450–500 kg) were divided into two groups of four horses each. The experimental group was fed RG (Korea Red Ginseng Powder Gold, 600 mg/kg/day) every morning for 3 weeks before the administration of furosemide, and the control group was not fed RG. All horses received water and food *ad libitum* during the experimental period. After 3 weeks, furosemide (Lasix) was administered intravenously as a single 0.5 mg/kg bolus to both groups of horses. Blood samples (10 mL) were collected from the jugular vein in heparin tubes at 0, 15, and 30 min, and 1, 2, 4, 8, and 24 h after administration. The collected blood was centrifuged at 3000 rpm for 5 min at 4°C, and the plasma supernatant was transferred to a 15 mL conical tube. The plasma was stored at −20°C until pretreatment. A sterile 24-F self-retaining catheter that was designed to remain in place was inserted into the bladder of a female horse and connected to a drainage bag to collect urine. The total volume of urine excreted was measured from 0 to 2 h after administering furosemide. When the drainage bag became full, the amount of excreted urine was measured using a graduated cylinder (5,000 mL) and replaced with another drainage bag.

A non-compartmental statistical model was used to determine the kinetic parameters of furosemide from equine plasma samples using the Phoenix WinNonlin Enterprise Program v5.3 (Pharsight Inc., St. Louis, MO, United States). The main parameters were the maximum plasma concentration (C_max_), elimination half-life (T_1/2_), area under the plasma concentration–time curve from time zero to the last sampling time (AUC_0−t_), area under the concentration–time profile extrapolated to infinity (AUC_0−∞_), mean residence time (MRT), total body clearance (CL), apparent volume of distribution (V_z_), and apparent volume of distribution at steady state (V_ss_). C_max_ was obtained directly from experimental data. T_1/2_ was calculated using the following equation: T_1/2_ = 0.693/λ_z_, where the elimination rate constant (λ_z_) is calculated from the terminal linear portion of the logged plasma concentration–time curve. AUC_0−t_ and AUC_0−∞_ were estimated using the log-linear trapezoidal rule. MRT was calculated from AUMC/AUC, where AUMC is the area under the first-moment curve. V_z_, V_ss_, and CL were obtained directly from the experimental data and processed using the WinNonlin software. The drug concentration versus time profiles were plotted using SigmaPlot (version 12.0). The pharmacokinetic parameters of the experimental groups were validated using Student’s *t*-test and considered statistically significant at *p* < 0.05 and *p* < 0.01. OPLS-DA analysis was conducted with MetaboAnalyst version 5.0 (McGill University, Ste. Anne de Bellevue, QC, Canada) (26). All data are expressed as the mean ± standard deviation (SD; *n* = 4).

## Results

### Method optimization and validation

For simultaneous analysis of furosemide, four ginsenosides (Rb1. Rb2, Rc, and Rd), and Digitoxin (Internal standard; IS), MRM transition, ionization polarity, precursor ion, product ion, fragmentor, and collision energy values were optimized. All analytes were eluted within a retention time of 3.0–3.5 min (Supplementary Table S1). The total runtime for analyzing one sample was 6 min, and multiple biological samples were rapidly analyzed consecutively using the Acquity HSS C18 column.

**Figure 1:**
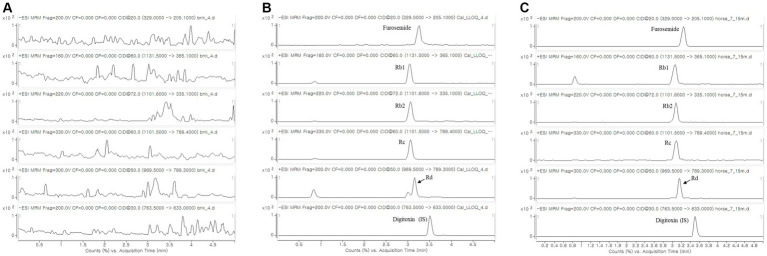
MRM chromatography of **(A)** blank equine plasma; **(B)** blank equine plasma spiked with furosemide, Rb1, Rb2, Rc, Rd (LLOQ), and digitoxin (IS, 500 ng/mL); and **(C)** equine plasma sample taken 15 min after 3 weeks of RG intake (600 mg/kg/day) and single injection of furosemide (0.5 mg/kg, IV). MRM, multiple reaction monitoring; LLOQ, lower limit of quantitation; IS, internal standard; RG, red ginseng.

The analytical method was validated by evaluating its specificity, linearity, Lower limit of quantitation (LLOQ), intra- and interday accuracy, intra- and interday precision, recovery, matrix effect, and stability. For specificity, the chromatograms of the blank equine plasma showed no interference from endogenous components, and furosemide and ginsenosides were detected without any issues (Figure 1). The linearity of the method was established for furosemide and ginsenosides in equine plasma, with a concentration range of 20–5,000 ng/mL and correlation coefficient of 0.9721–0.9933. The LLOQs of all the analytes were determined to be 20 ng/mL (Supplementary Table S2). Precision and accuracy were assessed using replicated experiments at four different concentrations. Intraday and interday accuracy was 98.3–111.2% and 93.3–110.4%, respectively. The intraday and interday precision was 0.7–12.5% and 2.9–9.4%, respectively (Supplementary Table S3). The extraction recovery was 91.7–109.4% and the total recovery was greater than 90% for all three sample concentrations. The matrix effect for all analytes was found to be 7.7–68.7%. Rb1 had a relatively high matrix effect, but no issues arose when analyzing its concentration in the equine plasma sample after RG intake. In the stability test, the stabilities of furosemide and ginsenosides were greater than 95.3%, and it was confirmed that they could be analyzed as stable samples for 7 days at −20°C (Supplementary Table S4).

### Quantification of RG components

When screening RG components from plasma collected from the experimental group prior to administering furosemide, eight PPD ginsenosides (Rb1, Rb2, Rc, Rd., CK, Rg3, F2, and Rh2) and three PPT ginsenosides (Re, Rg1, and Rh1) were monitored. Among them, four PPD ginsenosides (Rb1, Rb2, Rc, and Rd) were detected in the plasma and quantitatively analyzed (Table 1 and Figure 1). Rb1 showed a high concentration distribution in plasma as individual component, and PPDs were found to be distributed more predominantly in equine plasma than PPTs, which were not detected. These results, indicating differences from the control group, were further validated through OPLS-DA analysis comparing the control and experimental groups before and after furosemide administration at each blood collection time point. This analysis was based on the detected concentrations of RG as features, enabling the experimental group to infer the distribution of bioactive compounds from RG in the body (Supplementary Figure S2).

**Table 1:** Quantitative analysis of four ginsenosides in RG in equine plasma collected at 0, 15, and 30 min and 1, 2, 4, 8, and 24 h after single intravenous injection of furosemide (0.5 mg/kg) to eight horses.

Group	Horse	Ginsenoside (PPD, ng/mL)			
		Rb1	Rb2	Rc	Rd
Furosemide + RG	Horse #1	39.1 ± 22.9	33.1 ± 13.3	30.5 ± 6.4	27.6 ± 5.8
	Horse #2	262.1 ± 165.5	86.3 ± 40.7	158.2 ± 47.2	49.1 ± 16.3
	Horse #3	89.2 ± 46.8	34.0 ± 15.6	37.3 ± 14.0	23.1 ± 4.0
	Horse #4	97.4 ± 70.5	53.9 ± 36.7	30.1 ± 8.5	26.5 ± 7.8

The concentrations measured over time were calculated as the mean ± standard deviation. RG, red ginseng; PPD, (20s)-protopanaxadiol.

### Diuretic effect and pharmacokinetic interaction of furosemide

The volume of excreted urine was measured for 2 h to investigate the effect of RG on the diuretic action of furosemide. In the control group (*n* = 4), diuretic action was initiated immediately after administration, and after 5 min, a 2 L drainage bag filled with urine was replaced. In contrast, the volume of urine excreted by the experimental group was low until 30 min, after which it increased (*n* = 4). Owing to experimental limitations, the total urine volume after 2 h could be measured only in two animals per group. No significant difference in the total urine volume was observed after 2 h, even though the experimental group showed late diuresis (control group: 6.1 and 5.7 L; experimental group: 5.6 and 6.8 L).

**Figure 2:**
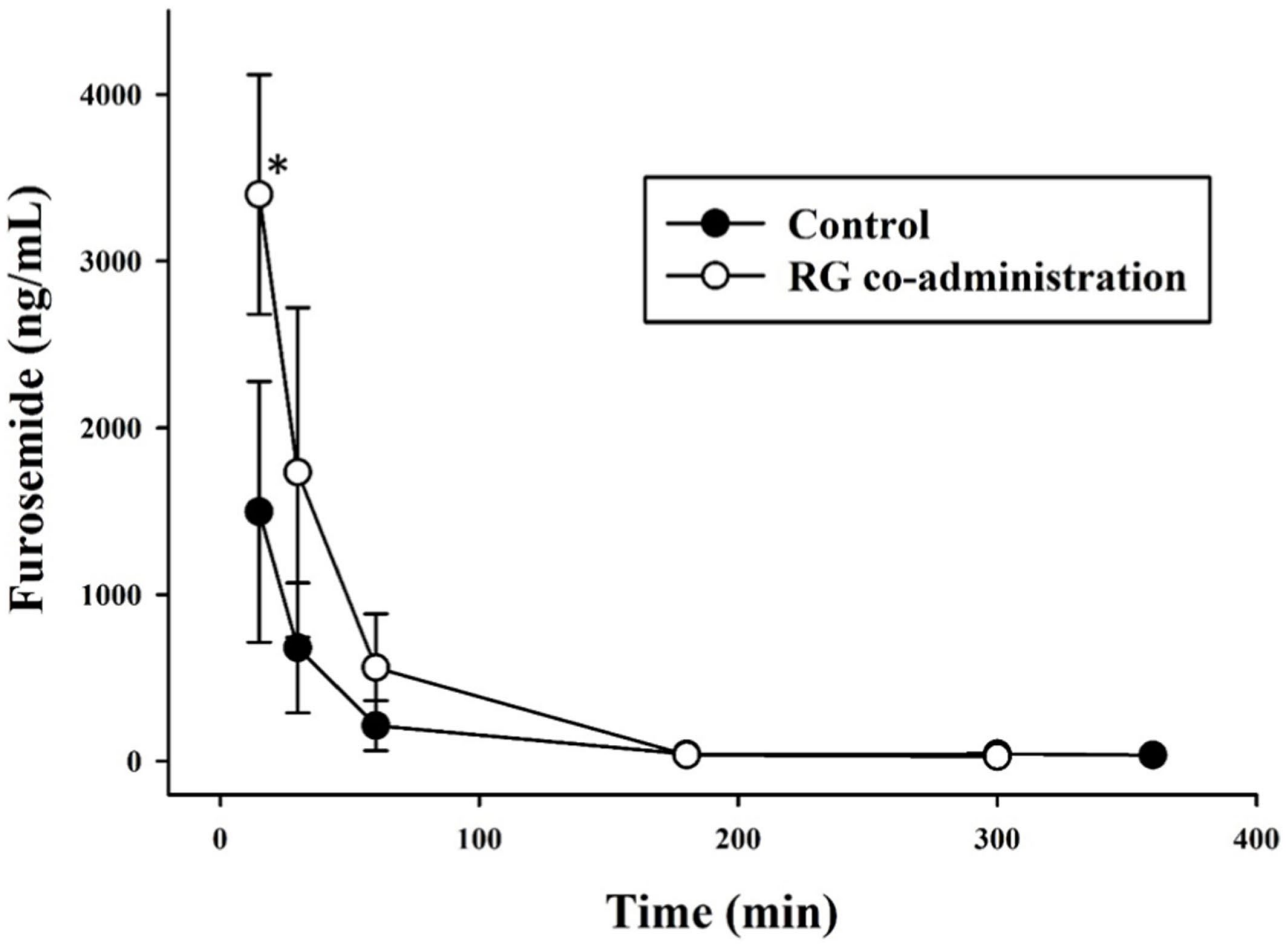
Furosemide concentration in plasma versus time for control (*n* = 4; furosemide, 0.5 mg/kg IV, black circles) and experimental groups (*n* = 4; RG, 600 mg/kg/day for 3 weeks; furosemide, 0.5 mg/kg IV, white circles). *p < 0.05, IV, intravenous; RG, red ginseng.

The time–concentration profile of furosemide in equine plasma after a single intravenous injection of furosemide and after 3 weeks of RC intake followed by a single intravenous injection of furosemide are shown in Figure 2. The pharmacokinetic parameters evaluated in the control and experimental groups and those calculated using non-compartmental statistical model are shown in Table 2. The C_max_, AUC_0−t_, and AUC_0−∞_ in the experimental group were significantly increased (*p* < 0.05) compared with those in the control group, and CL, V_z_, and V_ss_ were decreased (*p* < 0.05, *p* < 0.01).

**Table 2:** Pharmacokinetic parameters of furosemide in equines (*n* = 4 in each group) after intravenous administration of furosemide (0.5 mg/kg).

Pharmacokinetic parameter	Treatment	
	Control	Furosemide + RG (600 mg/kg/day)
C_max_ (ng/mL)	1,496 ± 783	3,400 ± 719*
AUC_0−t_ (h·ng/mL)	1,420 ± 790	3,481 ± 870*
AUC_0−∞_ (h·ng/mL)	1,511 ± 867	3,509 ± 872*
T_1/2_ (h)	1.8 ± 1.5	0.6 ± 0.1
MRT (h)	0.8 ± 0.4	0.5 ± 0.2
CL (mL/h/kg)	132 ± 60	46 ± 13*
V_z_ (mL/kg)	240 ± 85	44 ± 17**
V_ss_ (mL/kg)	95 ± 19	24 ± 13**

Data are presented as the mean ± standard deviation (*n* = 4). *p < 0.05, **p < 0.01. RG, red ginseng.

As a result of calculating the pk parameter for furosemide in the control and experimental groups, significant results were obtained for several parameters. C_max_, AUC_0−t_, and AUC_0−∞_ values in the experimental group increased significantly (*p* < 0.05) compared with those in the control group, and CL, V_z_, and V_ss_ decreased (*p* < 0.05, *p* < 0.01). These results indicated that not only the AUC of furosemide increased but also the clearance rate of furosemide decreased in horses regularly consuming RG.

## Discussion

In this study, we examined the pharmacokinetic interaction between furosemide and RG in equine plasma. To measure the primary bioactive compound of RG, ginsenoside, in plasma after the administration of RG and furosemide, we employed an analytical method utilizing LC–MS/MS, which was both utilized and validated.

In the experiment, horses have been fed RG for 3 weeks, but it has been necessary to confirm that the RG constituents remained in the body and bloodstream in the experimental group, unlike the control group. Ginsenosides are the main active substances in RG and have been widely known to have effects on the body in many literatures (6, 9, 13). Therefore, by detecting ginsenosides, it has been possible to assume that red ginseng components were distributed in the body of the experimental group.

In previous papers, several methods were introduced for the analysis of ginsenosides in plasma, including solid-phase extraction and time-of-flight mass spectrometry detection. The plasma protein precipitation extraction method employed in this study was relatively straightforward, potentially reducing experimental errors, and the analysis utilized the sensitive MRM detection method in tandem mass spectrometry (27, 28). Using validated method, we detected four PPD ginsenosides, namely Rb1, Rb2, Rc, and Rd., in plasma, with Rb1 exhibiting a high concentration distribution as individual components. Comparing these findings with previous research can be difficult, as there have been limited studies investigating ginsenosides in equine plasma. Nevertheless, the results align with prior human studies (29) in that PPDs (Rb1, Rb2, Rc, Rd., and CK) were predominantly identified, suggesting sufficient distribution of RG components in the plasma of the RG-fed group over a span of 3 weeks.

In several pharmacokinetic parameters of furosemide, significant changes were observed in both the control and experimental groups. Thus, AUC of furosemide increased, while the clearance rate of furosemide decreased in horses that regularly consumed RG. This suggests an elevated retention of furosemide within the body. Pharmacokinetic interactions can occur by affecting drug absorption, distribution, metabolism, and excretion. The mechanisms controlling the elimination of furosemide involve renal and hepatic glucuronidation metabolism, as well as the clearance via multidrug resistance protein 4 (MRP4) and organic anion transporters (OATs) transporters (30–32). These mechanisms could potentially be influenced by ginsenosides. For instance, it has been reported that Rb1’s concentration-dependent inhibition of UGT1A9 metabolic activity, competitive and non-competitive inhibition of UGT1A7 and 2B7 by Rg3, and inhibition of UGT1A1 and UGT2B7 reactions by PPT-type ginsenosides have been observed (33–36). According to papers by Jeon et al. and Seong et al., PPD-type ginsenosides (Rb1, Rb2, Rc, Rd., Rg3, CK, and Rh2) act as OATs inhibitors by inhibiting the activities of OATP1B1 and OATP1B3 (33, 37).

The diuretic effect of furosemide typically subsides within 2–3 h after intravenous administration (38). In our study, when we measured the diuretic response following furosemide administration, there was no noticeable difference in urine volume during the initial 2 h period between the control group and the group administered with RG. A previous human study reported an inhibition of furosemide’s diuretic effect due to RG intake (8), raising suspicions about the potential role of germanium inclusion in the RG manufacturing process as a contributing factor. However, a study conducted in rats found that the quantity of germanium present in RG did not result in a reduction of furosemide’s diuretic effect (39). This discrepancy may be attributed to differences between humans and animals. Nonetheless, further research is required to reach definitive conclusions.

The dietary composition and feeding management of horses should be given significant consideration, as they can profoundly impact factors such as nutrient absorption, digestion, and, consequently, both health and productivity (40, 41). RG is one of the frequently used supplements in the Asian and North American equine industries for purposes such as stress amelioration, disease prevention and performance improvement (42, 43). However, the absence of on-site nutritional guidelines on RG administration raises concerns about its usage. Particularly, there have been no studies conducted on the potential interactions between RG and diuretics in horses consuming RG, especially in specific conditions such as volume-related ailments and EIPH, where diuretics like furosemide need to be administered. The pharmacokinetic changes of furosemide observed in horses hold clinical significance, as they may lead to unforeseen drug side effects. These concerns highlight the need for industry and medical guidelines on the use of RG in the equine sector, to prevent economic and medical harm resulting from the overfeeding of RG. Furthermore, the research findings regarding RG interactions with drugs in horses can serve as foundational data for evaluating drug safety and considerations in drug development, as well as the animal pharmaceutical industry.

RG has been used as an herbal supplement in complementary and alternative medicine for thousands of years (44). However, there are still no clear guidelines regarding the optimal dose and duration of administration. Previous studies that evaluated the clinical effect of RG in humans varied the dose from 100 to 600 mg/kg and the duration of administration from 1 day to 27 months (45). The duration of administration for 2–4 weeks was commonly observed in other studies on herb-drug interactions (46). Considering the previous findings and available resources for the experiment, we administered RG to horses at a dose of 600 mg/kg for 3 weeks. However, further research is needed to analyze various responses and effects of RG depending on its concentration and duration of administration.

This study is the first research to analyze the herb-drug interactions between RG and drug in horses. However, this study also has some limitations. We used a total of eight female horses in the experiment, which can be considered a small sample size. Conducting experiments with horses is resource-intensive and expensive compared to other animal species, and limitations exist regarding the use of a large number of animals. Due to the limited number of experimental animals, the horses were divided into only two groups. With a larger number of animals available, we could have further subdivided the experimental groups, allowing for a more specific characterization of the effects of RG. Additionally, we aimed to minimize variables related to gender by using only one gender. We selected female horses for the experiment because quantifying urine output using a catheter in female horses following furosemide administration is feasible. In future large-scale studies, expanding the sample size and including both genders will provide a more comprehensive understanding of the responses. Furthermore, while this study primarily focused on the pharmacokinetic changes of furosemide, it may be necessary to include a comparison with the RG control group when monitoring its diuretic effect.

## Conclusion

In the present study, regular consumption of RG by horses led to significant alterations in the pharmacokinetic parameters of furosemide. These changes were marked by an increase in the AUC and a reduction in clearance. These findings suggest the potential involvement of ginsenosides from RG in modulating the metabolism or excretion of furosemide. Despite these pharmacokinetic changes, it’s important to note that there were no observed changes in the efficacy of furosemide. This underscores the complexity of pharmacokinetic interactions and highlights the need for further research to fully elucidate the mechanisms underlying these effects.

## Data availability statement

The original contributions presented in the study are included in the article/Supplementary material, further inquiries can be directed to the corresponding authors.

## Ethics statement

The animal study was approved by Korea Racing Authority (AEC-2307). The study was conducted in accordance with the local legislation and institutional requirements.

## Author contributions

YK: Conceptualization, Data curation, Formal analysis, Funding acquisition, Investigation, Methodology, Project administration, Resources, Software, Supervision, Validation, Visualization, Writing – original draft, Writing – review & editing. EL: Formal analysis, Methodology, Validation, Writing – original draft. HC: Funding acquisition, Methodology, Resources, Writing – original draft. TP: Funding acquisition, Methodology, Writing – original draft. AK: Funding acquisition, Methodology, Writing – original draft. JK: Resources, Writing – original draft. JY: Conceptualization, Formal analysis, Funding acquisition, Investigation, Methodology, Project administration, Resources, Writing – original draft, Writing – review & editing. HY: Conceptualization, Data curation, Funding acquisition, Methodology, Project administration, Resources, Supervision, Writing – original draft, Writing – review & editing.
