# Supplementary material for: A pharmacokinetic study on red ginseng with furosemide in equine
Source: Front Vet Sci. 2023 Nov 24;10:1319998. doi: 10.3389/fvets.2023.1319998 (PMC10704239; doi:10.3389/fvets.2023.1319998)
Supplement: Supplementary file 1 [file Data_Sheet_1.docx]

**Supplementary data**

**Materials**

Korea Red Ginseng Powder Gold manufactured by the Pocheon Ginseng Farming Association (Republic of Korea) was the RG powder used in this study. The total amount of ginsenosides, namely Rg1, Rb1, and Rg3, was 8 mg/g in this product. The standard material of ginsenosides (>98%) used for analysis was purchased from the Ambo Institute (Daejeon, Republic of Korea). Furosemide was purchased from the US Pharmacopeia (North Bethesda, MD, USA). Digitoxin was obtained from Sigma-Aldrich (St. Louis, MO, USA). HPLC-grade ACN and HPLC-grade DW were purchased from J.T. Baker (Phillipsburg, NJ, USA). FA was purchased from Junsei Chem (Chou-ku, Japan).

**Preparation of standard solutions**

Stock solutions of furosemide, ginsenoside, and IS were prepared in ACN at 100 mg/mL each and stored at -20°C until use. The internal standard solution was diluted with ACN to prepare a working standard at a final concentration of 25 µg/mL. The other stock solutions were each diluted with ACN and mixed with furosemide and ginsenosides to prepare working standard solutions with final concentrations ranging from 1 to 100 µg/mL. The working standard solutions (10 µL) were added to equine plasma (500 µL) to yield calibration standards of 20, 100, 200, 500, 1000, and 5000 ng/mL. QC samples were prepared using working solutions at final concentrations of 50, 400, and 2000 ng/mL.

**Protein precipitation of plasma**

IS working solution (2 µL) was added to the plasma sample (100 µL). Plasma proteins were then precipitated with 300 µL of ACN and vortexed for 30 s. Subsequently, the mixture was centrifuged at 135,000 rpm for 5 min. The supernatant was then transferred to a vial for LC-MS/MS analysis.

**Method validation**

The specificity of the method was determined by comparing the chromatograms of ten blank equine plasma samples to eliminate the possibility of interference from endogenous substances. A calibration curve was generated using six concentrations (20, 100, 200, 500, 1000, and 5000 ng/mL). The linearity of the curve was established by plotting the peak area ratio of the analyte to the internal standard against the nominal analyte concentration. The lowest level on the calibration curve was established as the LLOQ, which demonstrates good accuracy and precision (± 20%). The intraday precision and accuracy of the method were evaluated by measuring the concentrations of the QC samples (LLOQ at 50, 400, and 2000 ng/mL) three times in a single day, and the interday precision and accuracy were evaluated by measuring the concentrations of the QC samples over three consecutive days. Accuracy and precision are represented as the mean ± RSD. Extraction efficiency was evaluated by comparing the peak areas of the extracted spiked samples with those of the post-extracted spiked samples. The matrix effect on the analysis was determined by comparing the peak areas of the post-extracted spiked QC samples to those of the corresponding standard solutions. The stability of the method was evaluated by exposing the samples to -20°C for 7 days. The recovery, matrix effect, and stability tests were performed with three replicates at three different concentrations of the QC samples (50, 400, and 2000 ng/mL).

Table S1. RT, polarity, precursor ion, product ion, dwell, fragmentor, and CE evaluated through MRM.

| Compound | RT  (min) | Polarity  (+/-) | Precursor ion  (*m/z*) | Product ion  (*m/z*) | Dwell  (ms) | Fragmentor  (V) | CE  (V) |
| --- | --- | --- | --- | --- | --- | --- | --- |
| Furosemide | 3.2 | - | 329.0 | 205.1 | 150 | 200 | 20 |
| Rb1 | 3.0 | + | 1131.5 | 365.1 | 150 | 160 | 60 |
| Rb2 | 3.1 | + | 1101.6 | 335.1 | 150 | 220 | 72 |
| Rc | 3.1 | + | 1101.5 | 789.4 | 150 | 330 | 60 |
| Rd | 3.2 | + | 969.5 | 789.3 | 150 | 300 | 50 |
| CK | 4.2 | + | 645.4 | 203.0 | 150 | 200 | 40 |
| Rg3 | 3.5 | - | 829.5 | 783.5 | 150 | 220 | 22 |
| F2 | 3.5 | + | 807.4 | 627.4 | 150 | 250 | 30 |
| Rh2 | 4.4 | + | 587.4 | 407.4 | 150 | 200 | 20 |
| Rg1 | 3.0 | - | 845.5 | 799.5 | 150 | 200 | 30 |
| Rh1 | 3.2 | - | 637.4 | 475.4 | 150 | 200 | 30 |
| Re | 3.0 | + | 969.5 | 789.3 | 150 | 300 | 50 |
| Digitoxin (IS) | 3.5 | - | 763.5 | 633.0 | 150 | 200 | 30 |

Retention time (RT), collision energy (CE), multiple reaction monitoring (MRM), CK: Compound K, CE: Collision energy

Table S2. Calibration results in equine plasma

| Compound | Concentration (ng/mL) | Slope, A | Intercept, B | Correlation  coefficient, r^2^ |
| --- | --- | --- | --- | --- |
| Furosemide | 20‒5000 | 0.165601 | -0.011588 | 0.9921 |
| Rb1 | 20‒5000 | 0.115802 | -0.008549 | 0.9933 |
| Rb2 | 20‒5000 | 0.510931 | -0.056541 | 0.9860 |
| Rc | 20‒5000 | 0.194744 | -0.026617 | 0.9721 |
| Rd | 20‒5000 | 0.120490 | -0.015571 | 0.9816 |

* Slope and intercept refer to the regression equation y = Ax + B.

Table S3. Intra- and inter-day accuracy and precision (RSD) of the concentrations of furosemide and ginsenosides from equine plasma

| Compound | QC level | Nominal concentration  (ng/mL) | Intraday (n=3) | | Interday (n=3) | |
| --- | --- | --- | --- | --- | --- | --- |
|  |  |  | Accuracy (%) | RSD  (%) | Accuracy (%) | RSD  (%) |
| Furosemide | LLOQ | 20 | 105.9 | 2.1 | 107.9 | 7.9 |
|  | LQ | 50 | 96.3 | 2.7 | 98.0 | 3.0 |
|  | MQ | 400 | 96.3 | 2.3 | 100.8 | 7.0 |
|  | HQ | 2000 | 99.5 | 0.7 | 97.6 | 3.1 |
| Rb1 | LLOQ | 20 | 92.0 | 0.8 | 93.4 | 4.3 |
|  | LQ | 50 | 97.6 | 4.8 | 101.3 | 7.6 |
|  | MQ | 400 | 111.2 | 4.7 | 104.5 | 8.1 |
|  | HQ | 2000 | 98.5 | 3.0 | 101.2 | 5.1 |
| Rb2 | LLOQ | 20 | 105.7 | 4.3 | 108.1 | 4.2 |
|  | LQ | 50 | 98.0 | 4.4 | 104.9 | 5.8 |
|  | MQ | 400 | 107.3 | 10.7 | 105.1 | 8.6 |
|  | HQ | 2000 | 102.9 | 2.1 | 103.0 | 6.8 |
| Rc | LLOQ | 20 | 109.1 | 7.7 | 110.4 | 6.4 |
|  | LQ | 50 | 102.5 | 5.2 | 97.4 | 6.2 |
|  | MQ | 400 | 110.6 | 3.4 | 108.7 | 5.2 |
|  | HQ | 2000 | 103.7 | 2.7 | 105.8 | 6.6 |
| Rd | LLOQ | 20 | 113.3 | 2.0 | 112.1 | 2.9 |
|  | LQ | 50 | 98.3 | 12.5 | 102.5 | 9.4 |
|  | MQ | 400 | 105.9 | 5.7 | 103.7 | 5.6 |
|  | HQ | 2000 | 107.1 | 1.8 | 105.9 | 6.5 |

QC: quality control; LLOQ: lower limit of quantitation; LQ: low quality control; MQ: middle quality control; HQ: high quality control; RSD: relative standard deviation

Table S4. Matrix effect, recovery, and stability (each, n=3) of furosemide and ginsenosides in equine plasma

| Compound | QC level | Matrix effect ± RSD  (%) | Recovery ± RSD  (%) | Stability ± RSD  (-20℃, 7 days, %) |
| --- | --- | --- | --- | --- |
| Furosemide | LQ | 59.8 ± 13.1 | 100.2 ± 5.7 | 99.8 ± 6.5 |
|  | MQ | 57.2 ± 7.4 | 95.7 ± 5.0 | 98.1 ± 3.4 |
|  | HQ | 59.9 ± 1.1 | 99.1 ± 1.3 | 98.2 ± 4.0 |
| Rb1 | LQ | 7.7 ± 14.2 | 101.9 ± 10.5 | 93.0 ± 4.3 |
|  | MQ | 10.6 ± 11.9 | 101.8 ± 7.8 | 98.9 ± 7.6 |
|  | HQ | 9.5 ± 9.3 | 102.1 ± 8.5 | 95.8 ± 5.1 |
| Rb2 | LQ | 14.0 ± 12.7 | 91.7 ± 10.4 | 105.5 ± 1.4 |
|  | MQ | 17.8 ± 6.4 | 102.3 ± 2.9 | 109.6 ± 3.1 |
|  | HQ | 13.5 ± 3.6 | 102.2 ± 7.3 | 109.5 ± 10.9 |
| Rc | LQ | 12.4 ± 3.0 | 103.2 ± 2.5 | 107.3 ± 5.6 |
|  | MQ | 14.5 ± 10.8 | 102.7 ± 11.5 | 96.3 ± 0.7 |
|  | HQ | 14.4 ± 12.3 | 100.6 ± 3.6 | 106.9 ± 6.2 |
| Rd | LQ | 35.6 ± 3.6 | 98.0 ± 4.5 | 98.0 ± 2.7 |
|  | MQ | 36.4 ± 12.6 | 102.2 ± 4.4 | 98.3 ± 2.7 |
|  | HQ | 33.6 ± 6.9 | 109.4 ± 7.7 | 103.8 ± 3.0 |

QC: quality control; LLOQ: lower limit of quantitation; LQ: low quality control; MQ: middle quality control; HQ: high quality control; RSD: relative standard deviation


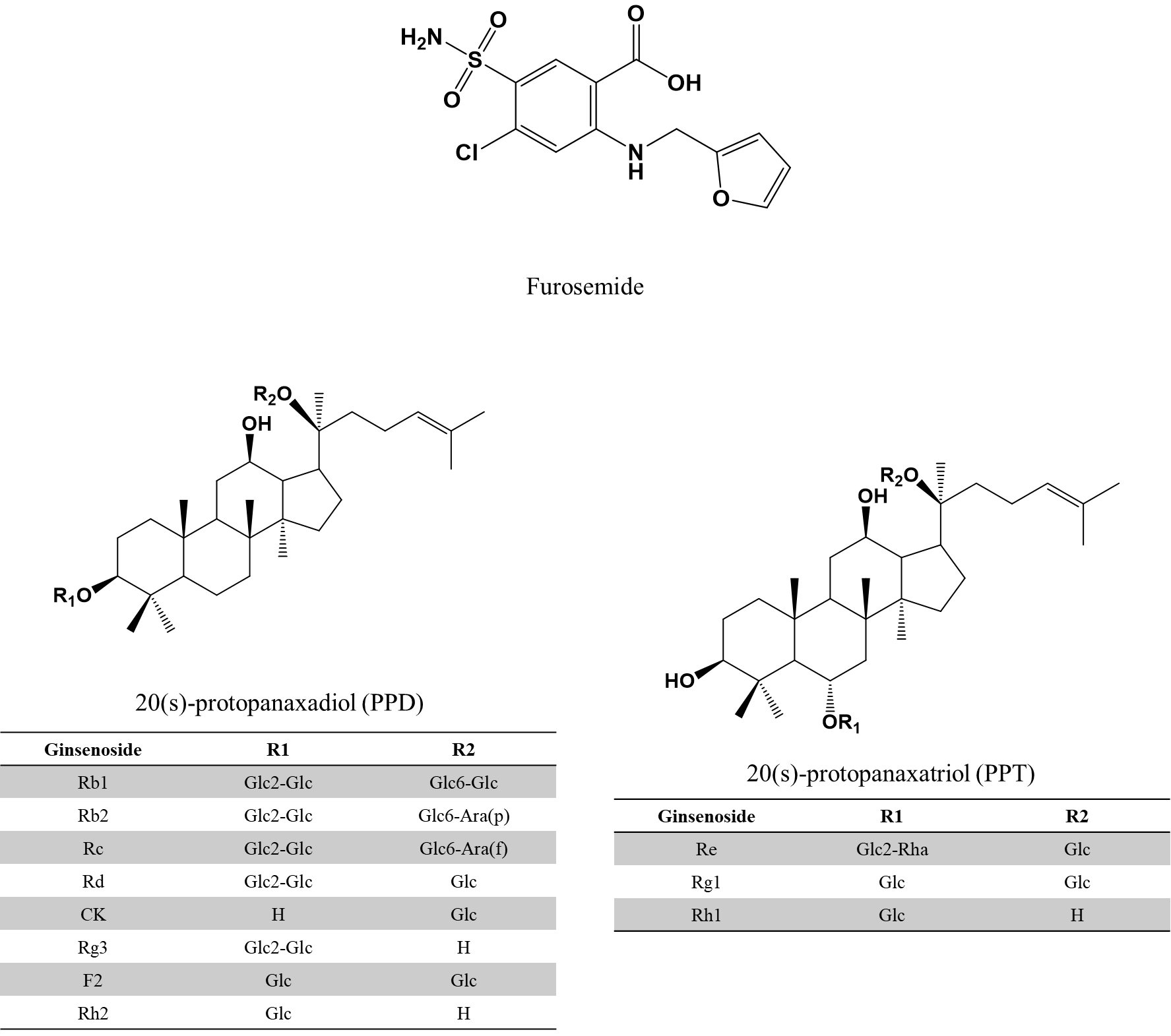


Fig. S1. Chemical structures of furosemide and ginsenoside


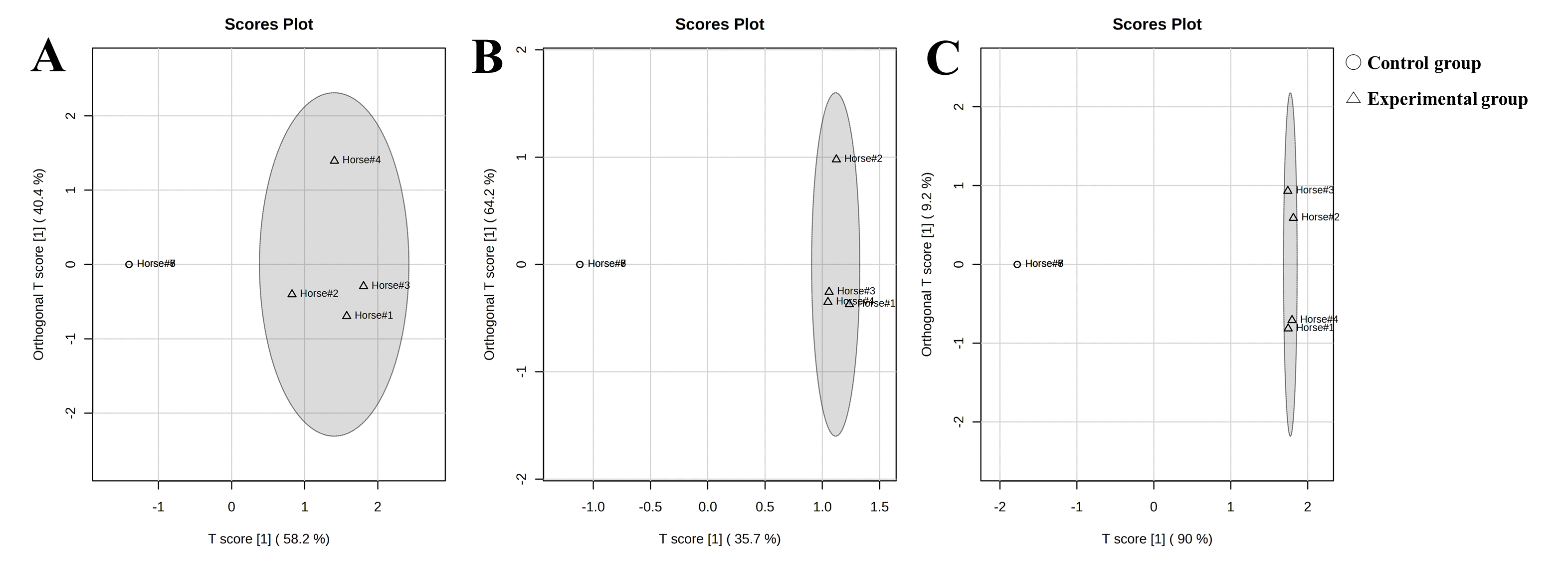


Figure S2. The representative results of OPLS-DA analysis for control group (white circles) and experimental group (white triangles) (A) before the injection of furosemide after RG intake (600 mg/kg/day) for 3 weeks (B) 4 h after the injection of furosemide (C) 24 h after the injection of furosemide. Abbreviations: RG: Red ginseng OPLS-DA: Orthogonal partial least squares discriminant analysis.
